# Supplementary material for: Identification of risk factors and development of a predictive model in patients using cefmetazole for international normalized ratio elevation
Source: PLoS One. 2025 Jul 28;20(7):e0322909. doi: 10.1371/journal.pone.0322909 (PMC12303349; doi:10.1371/journal.pone.0322909)
Supplement: Attachment — Submitted filename: PONE-D-24-16951_R1_Comments.pdf [file pone.0322909.s002.pdf]

Identification of risk factors and development of a predictive model in patients using cefmetazole for international normalized ratio elevation

Major comments

1. In abstract section, what group do you classify in patients with the predicted incidence probabilities of INR elevation ranging > 10 to < 30% and > 40 to 90%?

2. The definition of elevated INR in data collection section (93th – 94th line and 266th line) should be a ratio of maximum INR to baseline INR (maximum INR divided with baseline INR).

3. What is the full name of DOAC in data collection section (109th line)? If DOAC is direct oral anticoagulant, please revised the sentence "DOAC were patients with no dose changes for during and within 7 days prior to CMZ use."

4. Why does the p-value for the liver disorders in multivariate analysis in Table 1 reach 0.005, while their p-value in Table 2 reach only 0.05? Which is wrong?

5. In INR-elevated prediction model construction and accuracy verification section, what group do you classify in patients with score of 3, 5, and 8?

6. Why does patients with no-diabetes being the risk factors for CMZ-induced INR elevation? Your discussion proposed the clotting tendency of diabetes mellitus patients may have suppressed INR elevation in patients using CMZ, but healthy person without diabetes mellitus should not have coagulopathy. Please clearly explain in your discussion section.

7. Another limitation of your study is only Japanese patients were included, so this risk score model had have limited to generalizability in other races. Please add this limitation in your discussion section.

Minor comments:

1. In patient information part of materials and methods section, the dates of access to electronic medical record data in 73th-74th line should be used with the name of months instead of the number of months. It helps to reduce the confusion between month/date/year and date/month/year for readers.

2. Please add the full name of INR under Table 1.
