# Supplementary material for: Identification of risk factors and development of a predictive model in patients using cefmetazole for international normalized ratio elevation
Source: PLoS One. 2025 Jul 28;20(7):e0322909. doi: 10.1371/journal.pone.0322909 (PMC12303349; doi:10.1371/journal.pone.0322909)
Supplement: Attachment — Submitted filename: Response to Reviewers 20240806.docx [file pone.0322909.s001.docx]

***Responses to Reviewer #1***

We deeply appreciate the reviewers' comments and suggestions. We have revised the manuscript as suggested and ensured that it adheres to the required format. Our point-by-point responses to reviewers’ comments are provided below. Furthermore, all the changes made in the revised manuscript are highlighted in yellow.

**1)** **When submitting your revision, we need you to address these additional requirements.** **Please ensure that your manuscript meets PLOS ONE's style requirements, including those for file naming. The PLOS ONE style templates can be found at https://journals.plos.org/plosone/s/file?id=wjVg/PLOSOne_formatting_sample_main_body.pdf and**

**https://journals.plos.org/plosone/s/file?id=ba62/PLOSOne_formatting_sample_title_authors_affiliations.pdf**

Thank you for your comment. Accordingly, we added the following statements: “Identification of risk factors and development of a predictive model in patients using cefmetazole for international normalized ratio elevation”, “Yuta Yokoyama^1,3^ ^*^”, “^*^ Corresponding author”, and “E-mail: [yokoyama-yt@pha.keio.ac.jp](mailto:yokoyama-yt@pha.keio.ac.jp) (YY)”

Page 1 Lines 1–2, Page 1 Line 3, Page 1 Line 20, and Page 2 Line 22

All major sections were also modified to bold type, 18pt font and Sub-sections of major sections were modified to bold type, 16pt font.

Page 3 Line 26, Page 4 Line 47, Page 5 Line 70, Page 9 Line 140, Page 18 Line 221, Page 22 Line 300, Page 23 Line 307, and Page 24 Line 325 and Page 5 Line 71, Page 5 Line 90, Page 7 Line 118, Page 9 Line 141, Page 12 Line 168, Page 13 Line 182, and Page 17 Line 218

Furthermore, the abstract has been revised as required by the guidelines.

Pages 3 Lines 27–46

The position of tables and figures have also been revised in accordance with the guidelines.

Pages 10–11 Lines 150–167, Pages 15 Lines 211–214, Pages 16 Lines 215–217

Pages 9 Lines 148–149, Pages 13 Lines 196–198, Pages 14 Lines 203–206, Pages 14 Lines 208–210

Furthermore, we deleted " Address: 1-5-30 Shibakoen, Minato-ku, Tokyo 105-8512, Japan Tel.: +81-03-5400-2639; Fax: +81-03-5400-2651." and " Background, Methods, Results, Conclusions " and Author contributions.

Page 2 Lines 23–24 and Page 3 Lines 27, 33, 38, 44 and Page 23 Lines 314–324

**2) Thank you for stating the following in the Competing Interests section: "Dr. H. K. received research funding from Eli Lilly outside of the submitted work. Dr. K. M. received grants from Meiji Seika Pharma, Sumitomo Pharma and Shionogi outside of the submitted work. T.N. received research funding from Chugai, Daiichi Sankyo, Kyowa Kirin, Otsuka Pharmaceutical, Sanofi, and Shionogi outside of the submitted work. Conflicts that the editors consider relevant to the manuscript content have been disclosed."**

**We note that you received funding from a commercial source: "Eli Lilly, Meiji Seika Pharma, Sumitomo Pharma and Shionogi, Chugai, Daiichi Sankyo, Kyowa Kirin, Otsuka Pharmaceutical,and Sanofi".**

**Please provide an amended Competing Interests Statement that explicitly states this commercial funder, along with any other relevant declarations relating to employment, consultancy, patents, products in development, marketed products, etc.**

**Within this Competing Interests Statement, please confirm that this does not alter your adherence to all PLOS ONE policies on sharing data and materials by including the following statement: ""This does not alter our adherence to PLOS ONE policies on sharing data and materials.” (as detailed online in our guide for authors http://journals.plos.org/plosone/s/competing-interests).**

**If there are restrictions on sharing of data and/or materials, please state these. Please note that we cannot proceed with consideration of your article until this information has been declared.**

**Please include your amended Competing Interests Statement within your cover letter. We will change the online submission form on your behalf.**

Thank you for your comment. Based on the comments, we have added the following to the cover letter: “Although Dr. H. K, Dr. K. M, and Dr. T. N have received funding outside of the research, this does not alter our adherence to PLOS ONE policies on sharing data and materials. We have submitted to ICMJE.”

Cover letter

**3)** **In the online submission form, you indicated that "We do not make the data publicly available, but you can do so if you wish. Please contact the corresponding author."**

**All PLOS journals now require all data underlying the findings described in their manuscript to be freely available to other researchers, either 1. In a public repository, 2. Within the manuscript itself, or 3. Uploaded as supplementary information.**

**This policy applies to all data except where public deposition would breach compliance with the protocol approved by your research ethics board. If your data cannot be made publicly available for ethical or legal reasons (e.g., public availability would compromise patient privacy), please explain your reasons on resubmission and your exemption request will be escalated for approval.**

Thank you for your suggestion. We have made the data available in a public repository. You can access the data through the following private link URL: <https://figshare.com/s/a7cc966004fa34980f25>

**4) When completing the data availability statement of the submission form, you indicated that you will make your data available on acceptance. We strongly recommend all authors decide on a data sharing plan before acceptance, as the process can be lengthy and hold up publication timelines. Please note that, though access restrictions are acceptable now, your entire data will need to be made freely accessible if your manuscript is accepted for publication. This policy applies to all data except where public deposition would breach compliance with the protocol approved by your research ethics board. If you are unable to adhere to our open data policy, please kindly revise your statement to explain your reasoning and we will seek the editor's input on an exemption. Please be assured that, once you have provided your new statement, the assessment of your exemption will not hold up the peer review process.**

Thank you for your comment. We have published the data as mentioned above. We request you to review it.

**5) We note that you have included the phrase “data not shown” in your manuscript. Unfortunately, this does not meet our data sharing requirements. PLOS does not permit references to inaccessible data.**

**We require that authors provide all relevant data within the paper, Supporting Information files, or in an acceptable, public repository.**

**Please add a citation to support this phrase or upload the data that corresponds with these findings to a stable repository (such as Figshare or Dryad) and provide and URLs, DOIs, or accession numbers that may be used to access these data.**

**Or, if the data are not a core part of the research being presented in your study, we ask that you remove the phrase that refers to these data.**

Thank you for your comment. The text “data not shown” has been deleted as the data are now available on Figshare.

***Responses to Reviewer #2***

We really appreciate your time and effort in reviewing our manuscript and thank you for the insightful comments, constructive criticisms, and helpful advice. We have addressed the concerns raised by the reviewer and revised the manuscript to improve its overall readability.

**1)** **Between 2012 and 2017- Rewrite this as it does not seem appropriate to start a paragraph with this.**

Thank you for your comment. We have modified “Extended-spectrum β-lactamase-producing enterobacteria (ESBL-E) have become a major public health challenge owing to increasing number of patients affected by antimicrobial-resistant bacteria between 2012 and 2017, leading to increased morbidity and mortality rates [1,2].”

Page 4 Lines 48–50

**2) Why were minors not included in this study?**

Thank you for your confirmation. We excluded pediatric patients from the study owing to their increased vulnerability to vitamin K deficiency compared to adults, as well as the challenge of accurately diagnosing coagulopathy caused by CMZ in children. Various factors contribute to vitamin K deficiency in children, such as immature intestinal microflora and lower vitamin K production compared to adults, or low placental passage of vitamin K in neonates.

**3)** **IUPHAR/BPS - Kindly mention the fullform of this**

Thank you for your suggestion. Accordingly, we have modified “Drug and molecular target nomenclature conforms to the International Union of Basic and Clinical Pharmacology/British Pharmacological Society (IUPHAR/BPS) Guide standards”

Page 6 Lines 91–92

**4)** **Limit the discussion as it is very exhaustive and the deviating from the main reason of the study**

Thank you for your comment. As you pointed out, I have revised the discussion content and relevant references. The following sentence has been deleted.

“In this study, patients with an unstable baseline INR were excluded, and all patients with coagulopathy due to infections such as septic shock were excluded. Coagulopathy due to infection is caused by various factors, one of which is disseminated intravascular coagulation (DIC) [23]. D-dimer is generally used as an indicator of DIC [24], but at our institution it is not an item that is tested in general practice, and it is measured at the doctor's discretion if there is a concern about a coagulation abnormality. Since there was no difference between the two groups in patients for whom D-dimer was measured, it is thought that there was no coagulopathy due to infection.”

“Univariate analysis showed a statistically significant difference in the total dose of CMZ in INR-elevated group compared to non-INR-elevated group, but not a significant difference in daily dose. In the INR-elevated group, the median maximum INR during CMZ use was on day 3, compared to the INR non-elevated group, where the median maximum INR was observed on day 1. In this study, blood sampling was performed on arbitrary dates. The lack of significant difference in daily dose may be due to the difference in blood sampling date. The CMZ treatment period (period from the start of CMZ to the day of maximum INR) is also thought to be greatly affected by differences in blood sampling date. Therefore, the CMZ treatment period was not included in this analysis.”

“The reason for this may be due to the difference in blood sampling dates described above; the INR-elevated group received a higher dose, which may have affected the statistical significance of the AUC. In addition, AUC was considered to be the effect of other correlated factors and was excluded from the final model.”

“However, the number of patients who underwent bleeding was very small: 3 (4.9%) in the INR-elevated group and 3 (0.82%) in the INR-non-elevated group, so further research is considered necessary.”

“This means that the error in the INR measurement reagent is approximately 10%, and the increase is due to some factor other than measurement error (Manufacturer data reference).”

“23. Levi M, Keller TT, Van Gorp E, Ten Cate H. Infection and inflammation and the coagulation system. Cardiovascular Research. 2003. pp. 26–39. doi:10.1016/S0008-6363(02)00857-X”

“24. Adam SS, Key NS, Greenberg CS. D-dimer antigen: Current concepts and future prospects. Blood. American Society of Hematology; 2009. pp. 2878–2887. doi:10.1182/blood-2008-06-165845”

Page 18 Lines 225–230, Page 19 Lines 248–254, Page 19 Lines 256–258, Page 21 Lines 285–287, Page 21 Lines 288–290, Page 25 Lines 385–386, Page 25 Lines 387–388

Furthermore, the following sentence has been added to supplement the discussion.” This might be owing to differences in blood collection dates and other correlation factors and was excluded from the final model.”

Page 19 Lines 258–259

**5)** **Also elaborate regarding limitations of the study**

Thank you for your comment. As you pointed out, we have added the following sentence regarding the limitations of the study. “Furthermore, as this study was based on data from one city hospital and one university hospital in Japan, the sample size was limited to 429 patients. Therefore, it is considered that the influence of factors other than the identified risk factors has not been comprehensively evaluated. For further investigations, it is necessary to expand the sample size by including more survey facilities and using big data.”

Page 21 Lines 296–299

In addition, the following sentence has been deleted. “Further research is expected in the future for factors that have not been identified.”

Page 21 Lines 295–296

**The error is found in Page 3 Line 45.**

We deleted “,”

**Finally, we would like to express our gratitude for your comments and suggestions, which greatly helped us improve our manuscript.**
