# Supplementary material for: Identification of risk factors and development of a predictive model in patients using cefmetazole for international normalized ratio elevation
Source: PLoS One. 2025 Jul 28;20(7):e0322909. doi: 10.1371/journal.pone.0322909 (PMC12303349; doi:10.1371/journal.pone.0322909)
Supplement: Attachment — Submitted filename: Response_to_Reviewers.docx [file pone.0322909.s003.docx]

***Responses to Reviewer #1***

We deeply appreciate the reviewers' comments and suggestions regarding our manuscript. Our point-by-point responses to reviewers’ comments are provided below. Furthermore, all changes made to the revised manuscript are highlighted in yellow.

**Major comments**

**1)** **In abstract section, what group do you classify in patients with the predicted incidence probabilities of INR elevation ranging > 10 to < 30% and > 40 to 90%?**

Thank you for pointing this out. We have defined the probability of INR elevation based on the total score of each risk factor, referring to previous reports, so that we can classify all occurrence probabilities. We classified the probability of INR elevation into four categories: low risk (< 5%), medium risk (5–< 30%), high risk (30–< 90%), and very high risk (≥ 90%). The following passage was added to the method section:

Page 8 Lines 129–131, Reference 23

“From the total score of each risk factor, four risk classifications of low risk (< 5%), medium risk (5–< 30%), high risk (30–< 90%), and very high risk (≥ 90%) were performed for each elevated INR occurrence probability[21,23]”

Furthermore, we revised the description in each related section.

Page 3 Lines 37–38

“The predicted incidence probabilities of INR elevation were < 5% (low risk), 5–< 30% (medium risk), 30–< 90% (high risk), and ≥ 90% (very high risk). ”,

Page 13 Lines 182–184

“Risk scores between 0 and 2 were low risk (predicted probability < 5%), a risk score of 4 was medium risk (predicted probability 5–< 30%), those between 6–7 were high risk (predicted probability 30–< 90%), and those of 9 or higher were very high risk (predicted probability of occurrence ≥ 90%).”

Page 19 Lines 256–257

“In particular, patients possessing all of these risk factors are at high risk (probability of INR elevated: 30–< 90%) and may require INR monitoring.”

Table 3 has also been accordingly modified.

**2) The definition of elevated INR in data collection section (93th – 94th line and 266th line) should be a ratio of maximum INR to baseline INR (maximum INR divided with baseline INR).**

Thank you for pointing this out. We corrected the notation as follows:

Page 6 Lines 93–95

“Elevated INR was defined as a ratio of maximum INR to baseline INR (maximum INR divided with baseline INR) during CMZ use greater than 1.25.”

Page 20 Lines 276–278

“In this study, elevated INR was defined as a ratio of a ratio of maximum INR to baseline INR (maximum INR divided with baseline INR), during CMZ use, greater than 1.25.”

**3)** **What is the full name of DOAC in data collection section (109th line)? If DOAC is direct oral anticoagulant, please revised the sentence “DOAC were patients with no dose changes for during and within 7 days prior to CMZ use.”**

Thank you for your comment. In this study, direct oral anticoagulant (DOAC) was written as direct oral anticoagulants (DOACs) because there were multiple target drugs. We initially wrote ‘DOAC’ in the part you pointed out, but we will standardize it to DOACs to indicate multiple DOACs. The official name is written on lines 80-81, so we will correct this to DOACs.

Page 7 Lines 110–111

“DOACs were patients with no dose changes for during and within 7 days prior to CMZ use.”

In addition, there were also places in the discussion where the ‘DOAC’ was written, so we will correct it to ‘DOACs’.

Page 19 Lines 247–249

“However, since the effect on INR elevation varies depending on the type of DOAC, it is necessary to be careful when judging based on this result alone.”

**4) Why does the p-value for the liver disorders in multivariate analysis in Table 1 reach 0.005, while their p-value in Table 2 reach only 0.05? Which is wrong?**

Thank you for pointing that out. We checked the analysis data and corrected Table 2 because the *p-*value was 0.005.

Table 2 has been accordingly modified.

**5) In INR-elevated prediction model construction and accuracy verification section, what group do you classify in patients with score of 3, 5, and 8?**

Thank you for your comment. This time, the value of the β coefficient calculated from the results of the binomial logistic regression analysis was converted into a risk. As a result, warfarin use was scored as 5, and other factors were scored as 2. There were no patients with risk scores of 3, 5, and 8, in the case of 5, so the validity could not be evaluated, but it is possible to calculate from the regression equation assuming a patient with the risk factor of warfarin use only. When calculated under these conditions, the probability of INR elevation was estimated to be 20.9%. This probability of INR elevation is between the risk score of 4 and 6–7 points shown in Fig 2. Risk scores of 3 and 8 cannot be calculated as risk score values among the combinations of explanatory variables in the regression equation constructed this time, so the probability of INR elevation cannot be predicted. However, based on the above assumptions, it is assumed that the probability of occurrence is between 2 and 4 or 6–7 and 9 for risk scores of 3 and 8. Therefore, the following content has been included in the Discussion section:

Page 20 Lines 267–274

“In addition, since there were no actual patients, validity evaluation was not possible, but when assuming a patient with the risk factor of warfarin use only (risk score 5), the probability of INR elevation was calculated by regression equation to be 20.9%. This probability of INR elevation is between the risk score of 4 and 6–7 points shown in Fig 2. In this study, the identified risk scores were 2 for three factors and 5 for one factor, and due to issues with the combination of explanatory variables in the regression equation constructed, the probability of INR elevation could not be evaluated for risk scores of 3 and 8. However, based on the above assumptions, it is assumed that the probability of INR elevation is between 2 and 4 for a risk score of 3, and between 6–7 and 9 for a risk score of 8..”

**6)** **Why does patients with no-diabetes being the risk factors for CMZ-induced INR elevation? Your discussion proposed the clotting tendency of diabetes mellitus patients may have suppressed INR elevation in patients using CMZ, but healthy person without diabetes mellitus should not have coagulopathy. Please clearly explain in your discussion section.**

Thank you for pointing this out. As you pointed out, it is thought that coagulation disorders will almost never occur in all no-diabetes mellitus patients who use CMZ. It has been reported that diabetic mellitus patients are more susceptible to thrombosis than no-diabetes mellitus patients due to various factors, as described in the discussion. However, as a result of this risk score analysis, no-diabetes mellitus patients had a score of 2, and the probability of INR elevation in patients with a score of 2 is approximately 1%. A tendency was shown that the risk of INR elevation when combined with other risk factors such as liver disorder and nutritional risk. Therefore, we noted that not all no-diabetes mellitus patients develop coagulation disorders, and that caution is required when combined with other risk factors.

Page 19 Lines 237–240

“It should be noted that the risk score for no-diabetes mellitus patients is 2 points, and with a risk score of 2, the risk of INR elevation is approximately 1%, so not all no-diabetes mellitus patients will develop coagulation disorders when using CMZ. However, we think it would be prudent to use CMZ while keeping in mind that the INR may elevate when combined with other factors such as liver disorder and nutritional risk.”

**7)** **Another limitation of your study is only Japanese patients were included, so this risk score model had have limited to generalizability in other races. Please add this limitation in your discussion section.**

Thank you for pointing this out. As you have stated, this study only included Japanese patients, and it was not possible to study non-Japanese patients. For this reason, we have added the following passage to the section detailing the limitations of the study.

Page 21 Lines 284–286

“In addition, because the study only included Japanese subjects, it is unclear whether similar results would be obtained in non-Japanese patients.”

**Minor comments**

**1)** **In patient information part of materials and methods section, the dates of access to electronic medical record data in 73th-74th line should be used with the name of months instead of the number of months. It helps to reduce the confusion between month/date/year and date/month/year for readers.**

Thank you for pointing that out. We have corrected it to the name of months.

Page 5 Lines 73–75

“The dates of access to electronic medical record data were from June/1/2023 to July/31/2023, after obtaining approval from the ethics committee of each facility.”

**2)** **Please add the full name of INR under Table 1.**

Thank you for your comment. We included the full form of “INR, international normalized ratio” under Table 1.

Table 1 has been modified accordingly.

**Finally, we would like to express our gratitude to the reviewers for their comments and suggestions, which greatly helped us improve our manuscript.**
